# Conformation and Quantum-Interference-Enhanced Thermoelectric Properties of Diphenyl Diketopyrrolopyrrole Derivatives

**DOI:** 10.1021/acssensors.0c02043

**Published:** 2020-12-31

**Authors:** Renad Almughathawi, Songjun Hou, Qingqing Wu, Zitong Liu, Wenjing Hong, Colin Lambert

**Affiliations:** † Physics Department, Lancaster University, LA1 4YB Lancaster, United Kingdom; ‡ Beijing National Laboratory for Molecular Sciences, CAS Key Laboratory of Organic Solids, Institute of Chemistry, Chinese Academy of Sciences, Beijing 100190, China; § State Key Laboratory of Physical Chemistry of Solid Surfaces, iChEM, NEL, College of Chemistry and Chemical Engineering, Xiamen University, Xiamen 361005, China

**Keywords:** molecular electronics, diketopyrrolopyrrole (DPP) derivatives, quantum interference, thermoelectric properties, charge-transfer complex

## Abstract

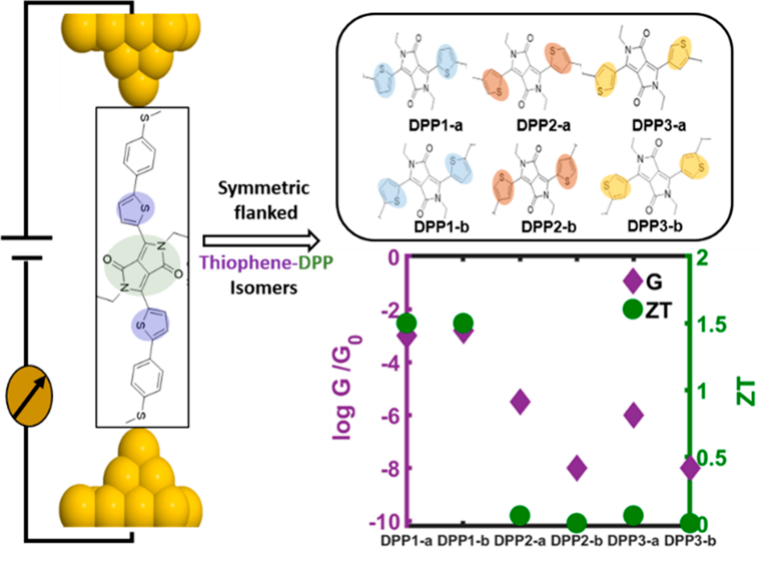

Manipulating the connectivity of external electrodes to central rings of carbon-based molecules in single molecule junctions is an effective route to tune their thermoelectrical properties. Here we investigate the connectivity dependence of the thermoelectric properties of a series of thiophene-diketopyrrolopyrrole (DPP) derivative molecules using density functional theory and tight-binding modeling, combined with quantum transport theory. We find a significant dependence of electrical conductance on the connectivity of the two thiophene rings attached to the DPP core. Interestingly, for connectivities corresponding to constructive quantum interference (CQI), different isomers obtained by rotating the thiophene rings possess the same electrical conductance while those corresponding to destructive quantum interference (DQI) show huge conductance variations upon ring rotation. Furthermore, we find that DQI connectivity leads to enhanced Seebeck coefficients, which can reach 500–700 μV/K. After including the contribution to the thermal conductance from phonons, the full figure of merit (*ZT*) for the CQI molecules could reach 1.5 at room temperature and it would further increase to 2 when temperature elevates to 400 K. Finally, we demonstrate that doping with tetracyanoquinodimethane can change the sign of the Seebeck coefficients by forming a charge-transfer system with the DPP.

The foundational experiments of Nongjian Tao^1^ and subsequent work exploring charge transport through single molecules connected to two metallic electrodes^2−4^ have led to the design of molecular-scale components such as switches,^4−6^ rectifiers,^7^ and highly conjugated molecular wires.^8^ A more recent goal of this research is the design of thermoelectric materials^9^ or devices^10^ based on single molecules or self-assembled monolayers,^11^ which can convert heat into electricity and contribute to the global challenge of green energy harvesting. Such organic materials and devices are potentially lightweight, flexible, environmentally friendly and cost-effective.^12,13^ Diphenyl diketopyrrolopyrrole discovered by Farnum et al. in 1974^14^ has unique properties, such as good conjugation, strong electron-withdrawing ability, thermal stability and photostability, and high-fluorescence quantum efficiency.^15,16^ It is widely used as a building block for organic molecules, both for fundamental studies of electronic properties and for industrial applications as dyes and pigments.^15,17^ Furthermore, the diketopyrrolopyrrole (DPP)-based molecule could be placed between aromatic rings such as the five-membered heterocycle thiophene,^18^ which creates the possibility of tuning their transport properties. In this work, stimulated by measurements of thermoelectricity in bulk DPP-based films, which show that a thermoelectric figure of merit of *ZT* = 0.25 could be achieved,^19^ we examine how room-temperature quantum interference in DPP cores influences their charge and heat transport properties and assess whether or not DPP derivatives are potential thermoelectric materials at the nanoscale.

Figure 1a shows the series of molecules of interest. DPP1, DPP2, and DPP3 contain thiophene rings with different connectivities, corresponding to inequivalent positions of the sulfur atoms of the thiophenes. Furthermore, each molecule is found to have two stable geometries labeled a or b, corresponding to different orientations of the thiophene rings relative to the DPP core. In what follows, we shall show that the Seebeck coefficients of DPP2 and DPP3 could reach 500–700 μV/K. After including the contribution of phonons to the thermal conductance, the full *ZT*s for molecules DPP1-a and -b reach 1.5 at room temperature and could increase to 2 when the temperature increases to 400 K. By exploring the effect of charge-transfer doping using tetracyanoquinodimethane (TCNQ), which is a well-known electron acceptor,^20,21^ we found that TCNQ can be used to change the signs of the Seebeck coefficients.

**Figure 1:**
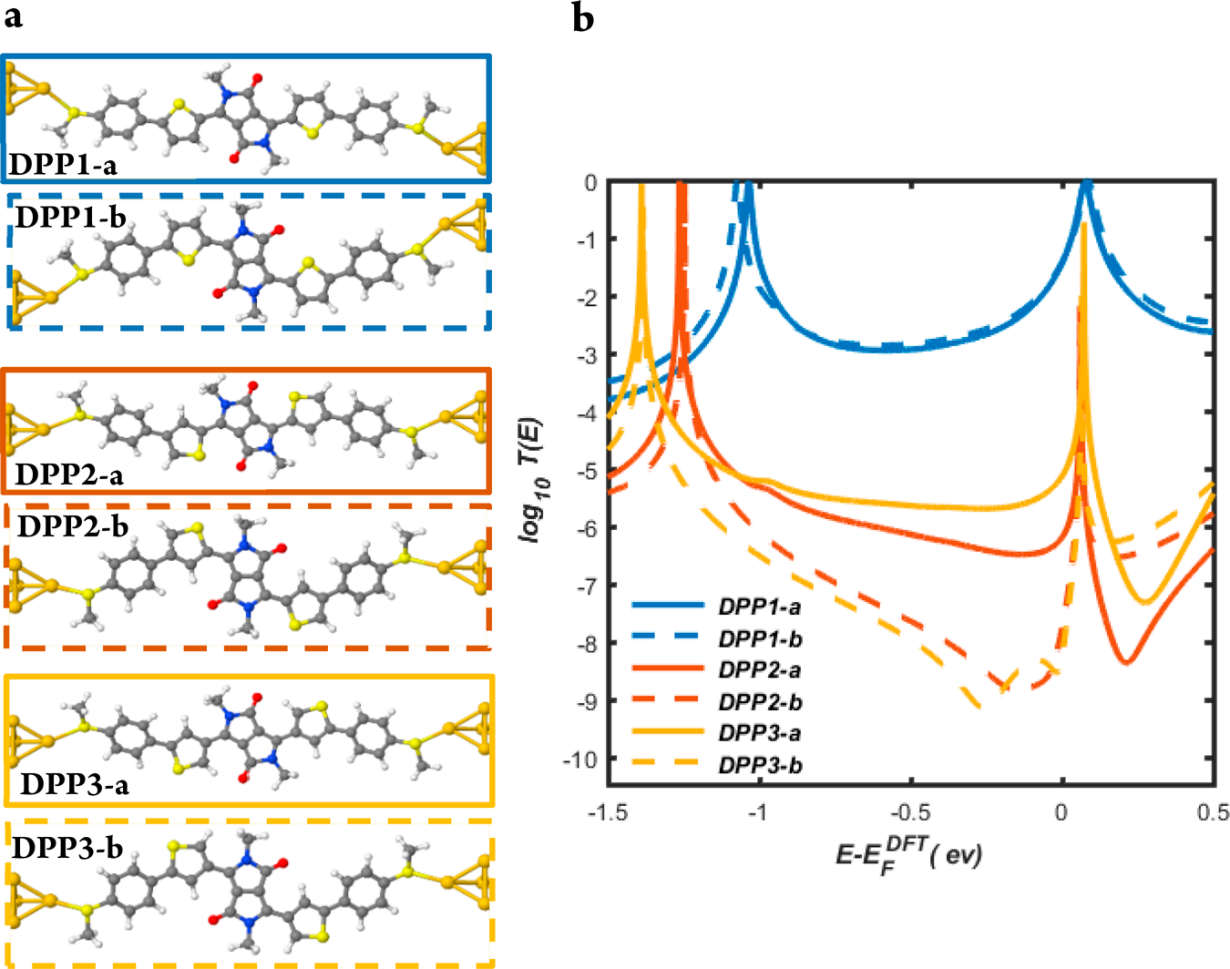
Charge transport properties of diketopyrrolopyrrole (DPP) derivative isomers attached to gold electrodes via -SMe anchor groups. (a) Models of the gold/molecule/gold sandwich junctions. The colors assigned to the atoms are as follows: gray for carbon, yellow for sulfur, red for oxygen, blue for nitrogen, white for hydrogen, and orange for gold electrode atoms. (b) DFT-based transmission spectra against Fermi energy (*E*_F_) in units of the quantum conductance *G*_0_ = 77μS. DPP1-a,-b (blue solid and dashed curves) exhibit constructive quantum interference (CQI) while DPP2-a,-b (red solid and dashed curves) and DPP3-a,-b (yellow solid and dashed curves) display destructive quantum interference (DQI).

## Results and Discussion

We systematically investigated the electrical and thermoelectrical properties of gold/thiophene-diketopyrrolopyrrole/gold hybrid junctions (Figure 1a) using quantum transport theory combined with the mean-field Hamiltonian of each geometry obtained from tight-binding binding models of each molecule (see more details in Methods). The DPP derivative isomers are defined through their connectivity and orientation of the thiophene rings. To reveal the effect of these features on transport properties, the a and b contact geometries between electrodes and molecules were fixed, and therefore the b geometry is obtained by rotating the linkers and the electrodes of a relative to the DPP core by 180° around the molecule axis. We find the b isomers display very similar transmission functions before and after their geometrical relaxation (see Figures S1 and S2 of the Supporting Information (SI)). Figure 1b shows that both DPP1-a and DPP1-b exhibit CQI, signaled by the absence of a dip in their transmission functions *T*(*E*) within the HOMO–LUMO gap, whereas both isomers of DPP2 and DPP3 exhibit DQI signaled by the presence of such a dip. In all cases, charge transport is mainly LUMO-dominated.

It is interesting to note quantum interference is sensitive to the dihedral angle between the central core and the neighboring thiophenes. To demonstrate this effect, we performed calculations, in which the two electrode–anchor–phenyl–thiophene substructures are rotated through a dihedral angle, θ, as indicated in Figure S8. Taking DPP2 as an example, panels b and c of Figure S8 show the total energy against θ and the corresponding transmission spectra arising for different values of θ. The two configurations (0° (DPP2-b) and 180° (DPP2-a)) have lower energies. The former is a global minimum, and the latter is a local minimum. By increasing θ from 0° (DPP2-b) to 180° (DPP2-a), the destructive quantum interference dip first moves to the right and then moves back. Therefore, the quantum interference pattern is indeed sensitive to rotations, with the mid-gap transmission decreasing by almost 4 orders of magnitude at the most energetically unfavorable angles. Rotation-angle sensitivities of this kind have been reported for other molecules in the literature, examples of which can be found in refs (31 and 32).

The transmission functions of molecules DPP1-a and DPP1-b, which exhibit constructive quantum interference (CQI; the blue solid and dashed curves) are rather similar. In contrast, the different isomers of molecules exhibiting destructive quantum interference (DQI) possess significantly different transmission functions. Near their transmission minima, the transmission coefficients of DPP2-a and DPP3-a (red and orange solid curves) are 2 to 3 orders of magnitude higher than those of DPP2-b and DPP3-b (red and orange dashed curves). These features in transmission functions could be interpreted from the perspective of the quantum interference between the molecular orbitals. From the molecular orbitals (MOs) of the gas-phase molecules DPP1, -2, and -3, Table S3 of the SI shows that the frontier molecular orbitals of DPP1-a,-b are delocalized across the molecule, while for DPP2-a,-b and DPP3-a,-b, the LUMO is localized on the DPP core. Consequently, the LUMO of these molecules will not contribute significantly to the transmission function. Furthermore, from the phases of the MOs on the terminal groups, it is clear that the LUMO+1 and HOMO will interfere destructively according to orbital product rule.^22,23^ In contrast, the delocalized LUMO of DPP1 interferes constructively with the HOMO, which leads to the higher transmission around Fermi energy. As indicated by the arrows in Tables S1 and S2, the LUMOs of DPP2-a and DPP3-a have a larger weight on the terminal groups than those of DPP2-b and DPP3-b, which contributes to the higher transmission coefficient within the HOMO–LUMO gap of DPP2-a,3-a compared with DPP2-b,3-b, shown in Figure 1.

**Figure 2:**
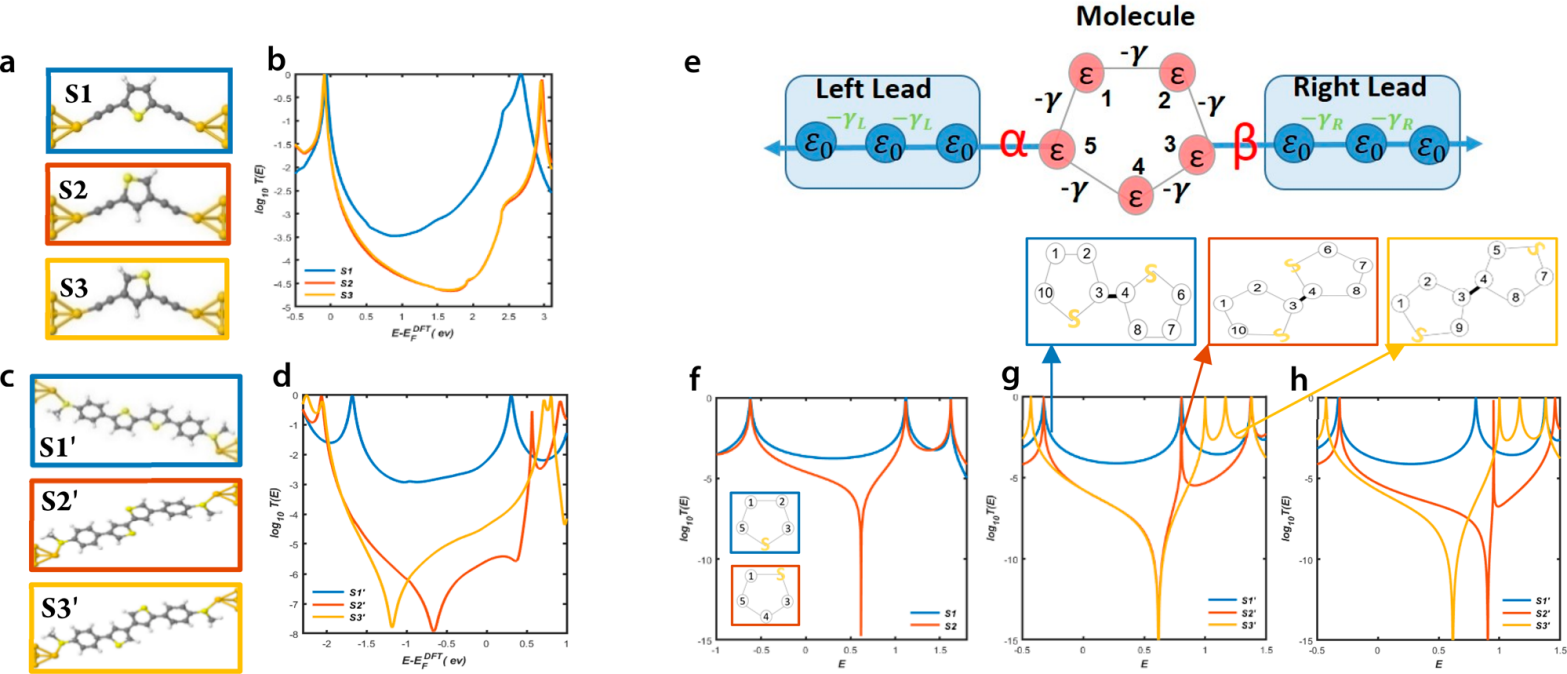
Comparison of DFT and tight-binding model-based transmission functions for three different connectivities of monomer and dimer thiophene rings. (a) Junctions formed from thiophene monomers S1, S2, and S3 with different connectivities. (b) Corresponding DFT-based transmission coefficients. (c) Junctions formed from thiophene dimers S1′, S2′, and S3′ with the connectivities corresponding to those of molecules DPP1-a, DPP2-a, and DPP3-a in Figure 1a. (d) The corresponding DFT-based transmission coefficients. (e) Tight-binding model (TBM) consisting of a five-membered ring attached to two semi-infinite one-dimensional chains through weak couplings α = β = 0.1. The on-site energies of the molecule (red dots) and the leads (blue dots) are ε and ε_0_, respectively. In the simplest model considered here, these are all set to zero except for those sites occupied by sulfur (see Methods), which are assigned an on-site energy *ε*_s_. For S1, S2, and S3, the sulfur sites are 4, 1, and 2, respectively, and the leads are connected to sites 3 and 5. The hopping integrals between nearest-neighbor atoms are set to −γ = −*γ*_L_ = −*γ*_R_ = −1. (f) TBM transmission functions for S1 and S2, obtained with a sulfur on-site energy of *ϵ*_s_ = −2. By symmetry, the transmission function of S3 is identical to that of S2. (g) TBM transmission functions for two thiophene rings with sites (10, 6) and (1, 7) connected to one-dimensional leads, respectively. The TB lattices associated with each curve are indicated by blue red and yellow arrows and correspond to the connections to the cores of S1′ to S3′ shown in panel c. For each connectivity, the on-site energies of both sulfurs have the same value (*ϵ*_s_ = −2). (h) As for panel g, except the on-site energies of both sulfurs of S2′ are changed to *ϵ*_s_ = −1.2, to account for the fact that their environments differ from those of S1′. This moves the curves closer to DFT results shown in panel d.

The difference between transmission functions of molecules exhibiting CQI or DQI is mainly attributed to the connectivity of the thiophene rings. In order to illustrate the dependence of the connectivity on transmission coefficients, it is helpful to understand the properties of central cores formed from thiophene rings alone. Panels a and c of Figure 2 show the transmission functions of cores formed from thiophene monomers and thiophene dimers with different connectivities. The transmission functions of molecules S2, S2′, S3, and S3′ in Figure 2b,d (red and yellow curves) exhibit DQI signaled by the presence of a dip in the *T*(*E*) within the HOMO–LUMO gap, whereas those of molecules S1 and S1′ in Figure 2b,d (blue curves) exhibit CQI, signaled by the absence of such a dip. The qualitative features of this connectivity dependence is captured by a simple tight-binding models (TBM), in which the π orbitals are assigned nearest-neighbor couplings only. Figure S3 of the SI shows the numbering system used to label atoms in these models.

The values used for the site energies of the sulfurs in the tight-binding model of Figure 2f,h are guided by our DFT calculations. Comparisons between the two approaches show that a TBM with only a single free parameter (i.e., the sulfur site energy, *ϵ*_s_) can capture the main qualitative features of the much more demanding DFT simulations. To illustrate the role of this parameter, Figure S3 shows how the TBM transmission coefficients would change if other nonoptimal values of *ϵ*_s_ are chosen. Results are shown for a series of on-site energies of the sulfurs, ranging from *ϵ*_s_ = −0.4 to *ϵ*_s_ = −2 and reveal that the transmission dip moves to lower energies as *ϵ*_s_ becomes more negative.

**Figure 3:**
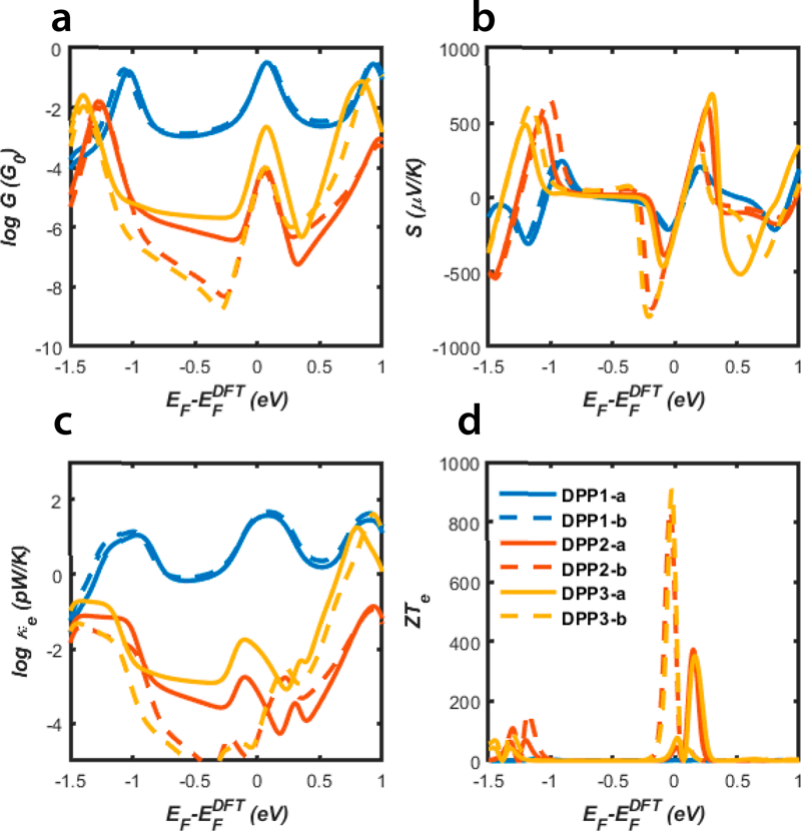
Thermoelectric properties of the thiophene-DPP isomers as the function of the Fermi energy at room temperature 300 K. (a) Electrical conductance *G*(*E*_F_). (b) Seebeck coefficients *S*(*E*_F_). (c) Thermal conductance *k*_*e*_(*E*_F_). (d) Electronic figure of merit *ZT*_*e*_(*E*_F_).

Starting from the DFT-based transmission functions, we evaluated thermoelectric properties of the above molecules (see Methods), including their electrical conductances, *G*; their Seebeck coefficients, *S*; electronic thermal conductances, *k*_*e*_, and electronic figure of merit, *ZT*_*e*_. These are shown in Figure 3. The Mott formula 
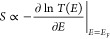
 indicates that a large Seebeck coefficient can be obtained if the Fermi energy (*E*_F_) happens to coincide with a steep slope of electron transmission coefficient, *T*(*E*).^24,25^ As a consequence, the Seebeck coefficient is higher for molecules exhibiting DQI (red yellow curves) as shown in Figure 3b. We find that the Seebeck coefficients for DQI molecules DPP2-a,-b and DPP3-a,-b could reach 400–700 μV/K. Figure 3c shows *k*_*e*_ due to the electrons obtained from the electron transmission functions (see Methods). The heat transport due to electrons for the CQI in Figure 3c (blue curve) has a shape similar to CQI of the electrical conductance in Figure 3a, reflecting the Wiedemann–Franz law. The thermal conductance due to electrons for DPP1-a,-b is in the range between 1 and 40 pW/K, which is comparable with typical thermal conductances due to phonons, ∼10 pW/K. The molecules exhibiting DQI possess substantially lower values of *k*_*e*_. Consequently, their high *ZT*_*e*_ do not lead to high values of the full *ZT*.

**Figure 4:**
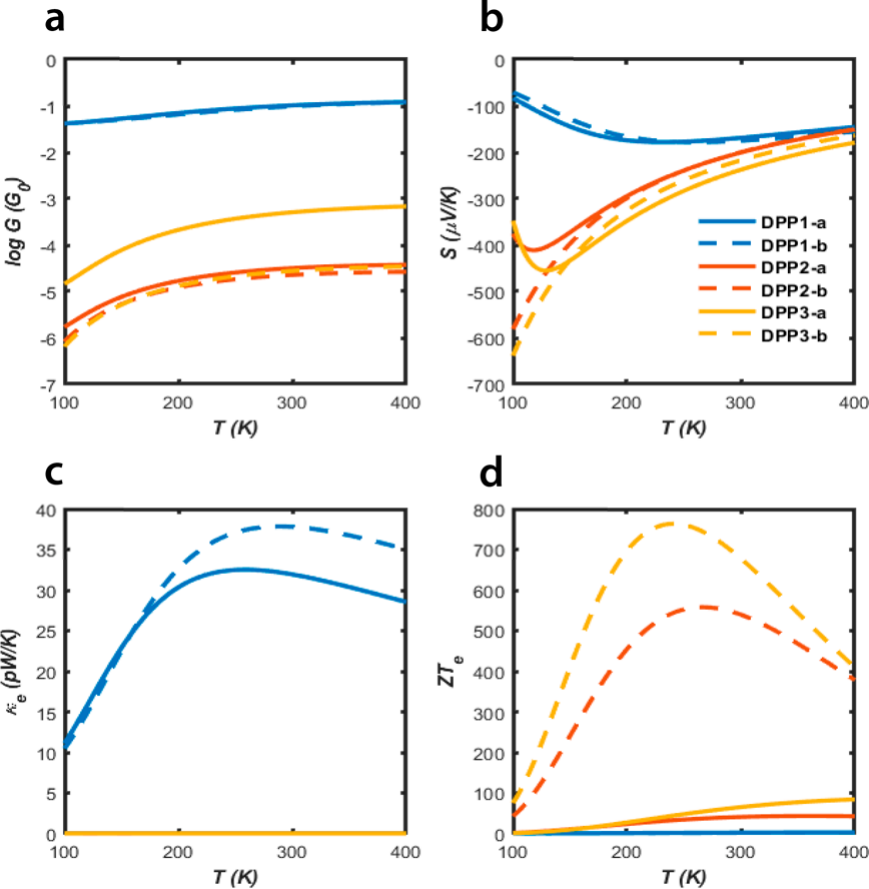
Thermoelectric properties of the molecules as a function of temperature at *E*_F_ = 0 eV: (a) Electrical conductance, *G*(*T*); (b) Seebeck coefficient, *S*(*T*); (c) thermal conductance, *k*_*e*_; (d) electronic figure of merit, *ZT*_*e*_(*T*).

Figure 4 shows the effect of temperature (*T*) on the thermoelectric performance of thiophene-DPP derivatives and reveals that *ZT*_*e*_(*T*) increases with temperature up to a maximum value, before decreasing at higher temperatures. As mentioned above, *ZT*_*e*_(*T*) only includes the thermal conductance *k*_*e*_.

**Figure 5:**
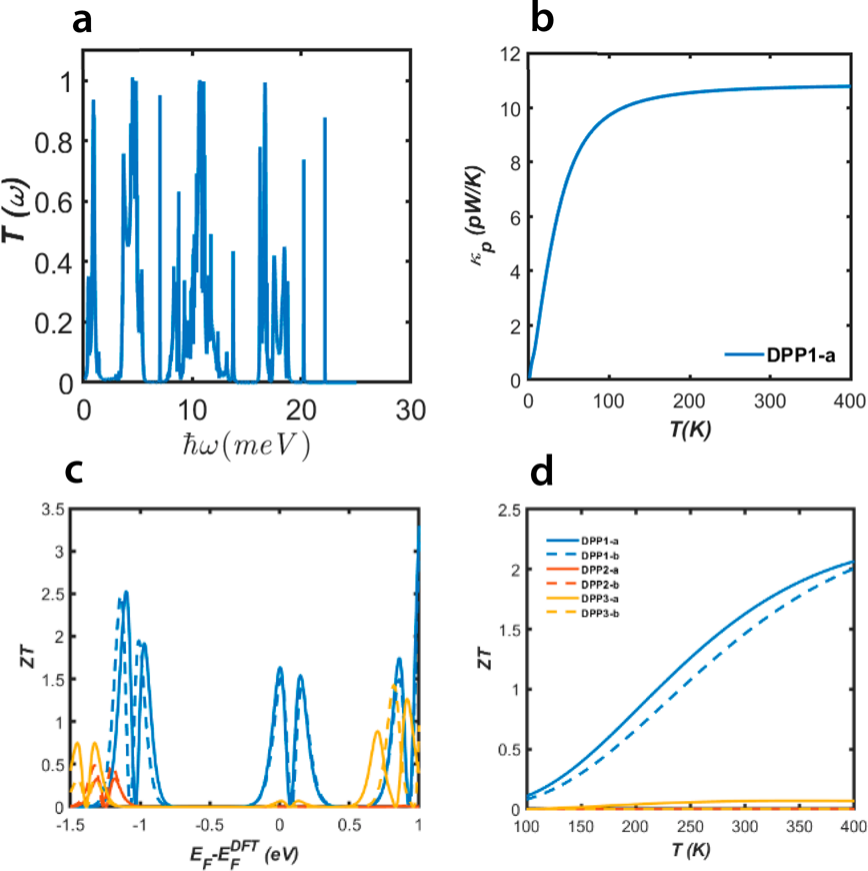
Thermoelectric properties of the molecules: (a) Phonon transmission function for DPP1-a; (b) phononic contribution to the thermal conductance for DPP1-a; (c) full *ZT* as a function of Fermi energy at room temperature 300 K for molecules shown in Figure 1a; (d) full *ZT* as a function of temperature for molecules shown in Figure 1a.

When the thermal conductance due to the phonons *k*_ph_ is included,^26^ the full *ZT* = *S*^2^*GT*/(*κ*_*e*_ + *κ*_ph_)^27^ and only this is physically relevant. In order to compute *k*_ph_ to the thermal conductance, we calculate the transmission coefficient of phonon *T*_ph_(*ℏω*) as a function of their frequency, ω (see Methods). Since the highest *ZT* occurs when *k*_ph_ is less than or comparable with *k*_*e*_, we focus initially on the highest conductance molecule DPP1-a, with its phonon transmission coefficient and thermal conductance due to the phonons being presented in Figure 5a,b. The phonon thermal conductances are found to be 16.9 and 8 pW/K for DPP2-a and DPP2-b, respectively (see Figure S6 of SI). For DPP1-a Figure 5c shows the room-temperature full *ZT* versus the Fermi energy and demonstrates a high room-temperature *ZT* ∼ 1.5 is achievable. Figure 5d shows the temperature dependence of *ZT* for all molecules and reveals that the *ZT* of DPP1 can reach ∼2 when the temperature increases to 400 K. However, the molecules exhibiting DQI show quite low values of the full *ZT*, because *k*_ph_ dominates the electronic contribution. DPP2-/3-b have the lowest electrical conductances and the highest Seebeck coefficients. *ZT*_*e*_ = *GS*^2^*T*/*κ*_*e*_, and therefore, if phonons are neglected and provided the Wiedemann–Franz law is valid (i.e.,  constant), the molecules with the highest *S* will have the highest *ZT*_*e*_. However, this means that the low-*G* molecules have low values of *k*_*e*_ and therefore after including the phonons the percentage increase in thermal conductance is greatest for low-*G* molecules. This is why molecules with high *ZT*_*e*_ have a low *ZT*. More quantitatively, the thermal conductance due to phonons is of order *κ*_ph_ = 8 pW/K, whereas for the low-*G* molecules DPP2-b and DPP3-b, *κ*_*e*_ ≈ 10^–4^ pW/K. Consequently, phonons dominate their thermal conductance and their full *ZT* is low. For the CQI molecules DPP1-a/-b, where *κ*_ph_ = 11 pW/K and *κ*_*e*_ = 32 pW/K, the full *ZT* is higher, because phonons have a much smaller effect on the thermal conductance.

Figure S5 of the SI shows that a similar temperature dependence is obtained if the Fermi energy deviates from the DFT-predicted value by −0.1 eV.

**Figure 6:**
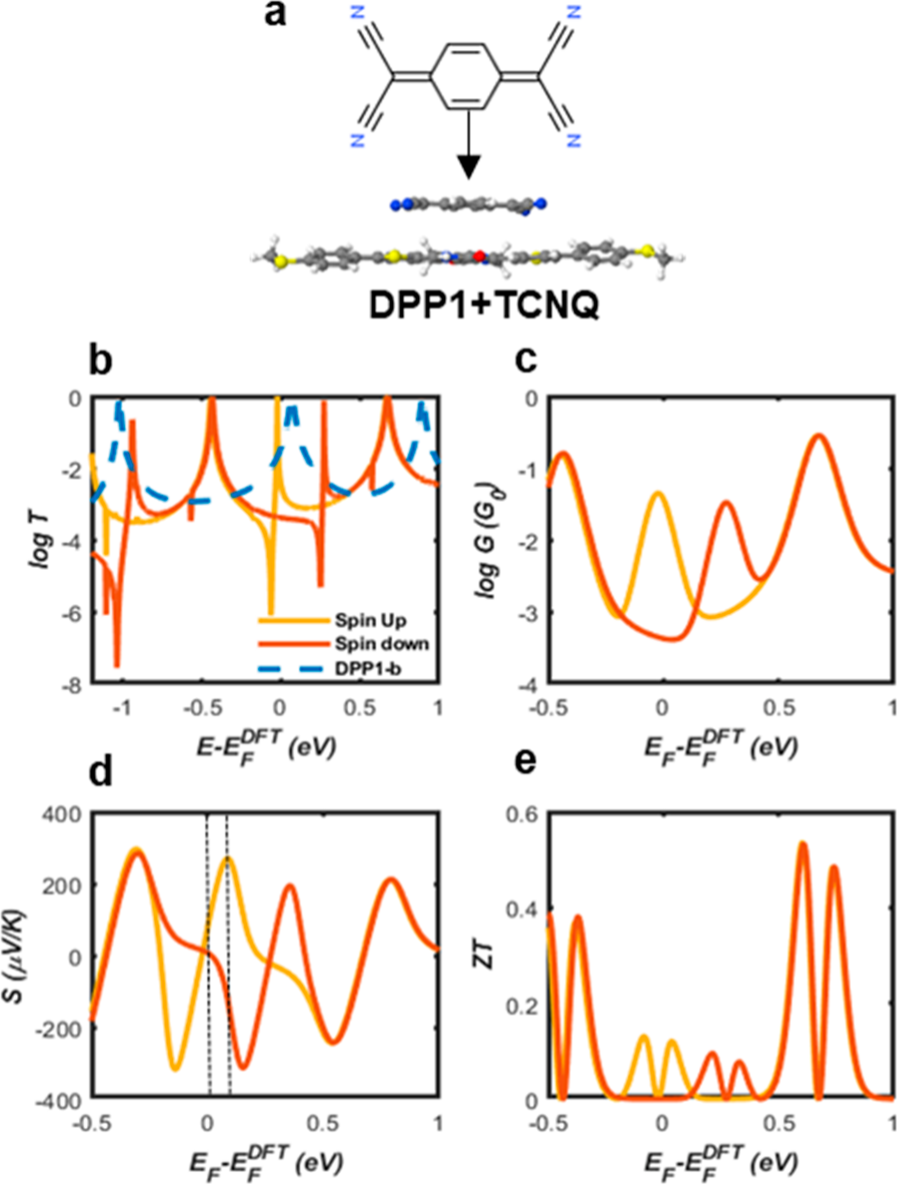
DFT-based transmission functions for DPP1-b + TCNQ: (a) Configuration of the system containing a single molecule DPP1 with TCNQ; (b) transmission coefficients against Fermi energy, *E*_F_ (blue curve, transmission functions of DPP1-b; red and yellow curves, spin up and spin down transmission functions of the donor–acceptor charge-transfer complex, respectively); (c) electrical conductance, *G*; (d) Seebeck coefficients, *S*; (e) room-temperature *ZT* versus Fermi energy.

Starting from the above molecular junctions, we consider the possibility of tuning their electrical and thermoelectrical properties, by doping with TCNQ^21,28^ to form charge-transfer complexes. Our aim is to investigate the influence of the presence of TCNQ acceptor molecule on the transmission coefficient of the DPP derivatives in Figure 1a. As an example, we chose DPP1, because the undoped molecule has the highest *T*. Starting from this high value, the aim is to determine if it is possible to increase *ZT* even further. DPP1-a and DPP1-b have the same central core and will bind to TCNQ in the manner. The results in Figure 6b show that TCNQ gains electrons from the backbone, which induces negative gating on the backbone DPP1-b. Consequently, spin polarized transport is observed due to the charge transfer from the DPP1-b to TCNQ. In addition, the two Fano-resonances are generated due to the weak coupling between the acceptor and donor, so that the acceptor behaves like a pendant group.^29,30^ Then, if we replaced sulfur atom with oxygen, there is no significant difference in the behavior of the transmission coefficient (Figure S7). For the DPP1-b+TCNQ complex, we computed the thermoelectric properties, the electrical conductance, the Seebeck coefficient and the full *ZT* by using an estimated *k*_ph_ value equal to 10 pW K^–1^. In the range between the two vertical dashed lines in Figure 6d, the Seebeck coefficients are positive, which indicates that the sign is tuned by TCNQ doping. The full *ZT* is around 0.1 which is suppressed compared to the undoped junction.

## Conclusion

On the basis of density functional theory and the quantum transport theory, the electron transport properties have been investigated for thiophene-DPP derivatives (DPP1, DPP2, and DPP3). This work illustrates that varying the position of the sulfur atom in thiophene rings has a significant influence on their electrical and thermoelectric properties. It is further verified by studying the connectivity of the two-thiophene-ring systems in the absence of DPP core. In addition, the rotation of the flanked rings could cause huge variations in the conductance when inserting the DPP core into the two-thiophene system. Furthermore, DQI molecules 2 and 3 systems show high Seebeck coefficients, which could reach 500–700 μV/K. After including the contribution from phonons to the thermal conductance, we found that, due to the presence of CQI, the full *ZT* of DPP1 reaches 1.5 at room temperature and could increase to 2 when temperature elevates to 400 K. Finally, we demonstrated that the Seebeck could be further tuned by introducing a TCNQ dopant which could gain electrons from DPP, leading to the sign change for the Seebeck coefficients even though DPP is a stronger acceptor. These results suggest that DPP derivatives are versatile materials for thermoelectric functions, whose performance can be tuned by varying their connectivity to electrodes, changing the positions of sulfur atoms and varying the orientation of their thiophene rings to obtain different isomers.

## Methods

### DFT Calculation

The optimized geometry and ground state Hamiltonian and overlap matrix elements of each structure were self-consistently obtained using the SIESTA^33^ implementation of density functional theory (DFT). SIESTA employs norm-conserving pseudopotentials to account for the core electrons and linear combinations of atomic orbitals to construct the valence states. The generalized gradient approximation (GGA) of the exchange and correlation functional is used with the Perdew–Burke–Ernzerhof parametrization (PBE)^34^ a double-ζ polarized (DZP) basis set, a real-space grid defined with an equivalent energy cutoff of 200 Ry. The geometry optimization for each structure is performed to the forces smaller than 10 meV/Å.

### Tight-Binding Model

The Hamiltonian of the simple tight-binding model describes a single orbital per atom with nearest-neighbor couplings γ = −1. All site energies are set to zero, except the site energies of sulfurs, which in Figure S3 were chosen to range from −0.4 to −2.

### Transport Calculations

The mean-field Hamiltonian obtained from the converged DFT calculation or a tight-binding Hamiltonian (using a single orbital energy site per atom with Hückel parametrization) was combined with our homemade implementation Gollum^35^ to calculate the phase-coherent, elastic scattering properties of each system consisting of left gold (source) and right gold (drain) leads and the scattering region (molecules DPP1, DPP2, and DPP3). The transmission coefficient *T*(*E*) for electrons of energy *E* (passing from the source to the drain) is calculated via the following relation:
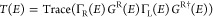
1In this expression, Γ_L,R_(*E*) = *i*(Σ_L,R_ (*E*) – Σ_L,R_^†^(*E*)) describes the level broadening due to the coupling between left (L) and right (R) electrodes and the central scattering region, Σ_L,R_(*E*) are the retarded self-energies associated with this coupling and *G*^R^(*E*) = (*ES* – *H* – Σ_L_ – Σ_R_)^−1^ is the retarded Green’s function, where *H* is the Hamiltonian and *S* is overlap matrix. Using obtained transmission coefficient *T*(*E*), the electrical conductance *G*, the Seebeck coefficient *S*, and the electronic thermal conductance κ_*e*_ and the electronic figure of merit *ZT*_*e*_ can be calculated through the following formula:
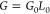
2
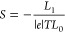
3
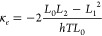
4
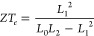
5In the linear response the quantity of Lorenz number *L*_*n*_(*T*,*E*_F_) is given by
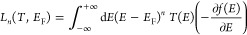
6where *G*_0_ = 2*e*^2^/*h* is conductance quantum, *e* is the charge of an electron; *h* is the Planck’s constant; *E*_F_ is the Fermi energy; *f*(*E*) = (1 + exp((*E* – *E*_F_)/*k*_B_*T*))^−1^ is the Fermi–Dirac distribution function, *T* is the temperature, and *k*_B_ = 8.6 × 10^–5^ eV/K is Boltzmann’s constant. The electronic figure of merit ignores *k*_ph_ due to phonons, whereas *ZT* experimentally is defined by *ZT* = *S*^2^*GT*/(*k*_*e*_ + *k*_ph_), which includes the thermal conductance due to both phonons and electrons in the denominator. To calculate the thermal conductance *k*_ph_ due to phonons, the force constant matrix, *K*, is obtained by finite differences:
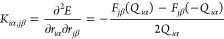
7where *E* is the total energy and *r*_*iα*_ (*r*_*jβ*_) is the displacement of atom *i*(*j*) in the coordinate direction α(β). The geometry is relaxed until the force of each atom is equal to 0.01 eV Å^–1^. By shifting each atom (*i*) with *Q*_*iα*_ = 0.01 Å in the direction α = *x*,*y*,*z* the forces on atom along each β = *x*,*y*,*z* direction, where *F*_*jβ*_ (*Q*_*iα*_) is calculated. Thus, the dynamical matrix *D* can be obtained by , where *m*_*i*_*m*_*j*_ are the masses of atom *i* and atom *j*. Then the dynamical matrix is used to compute the transmission probability of phonons using the Gollum transport code with eqn 1. The corresponding phonon thermal conductance is given by
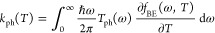
8where  is the Bose–Einstein distribution.

## Supporting Information Available

Details of DPP rotation geometries; thiophene connectivity; molecular orbital; charge transport properties; tight-binding model with more energy sites; thermoelectric properties calculation of molecular junctions (PDF)
