# Supplementary material for: Conformation and Quantum-Interference-Enhanced Thermoelectric Properties of Diphenyl Diketopyrrolopyrrole Derivatives
Source: ACS Sens. 2020 Dec 31;6(2):470–6. doi: 10.1021/acssensors.0c02043 (PMC8021221; doi:10.1021/acssensors.0c02043)
Supplement: Supplementary file 1 — se0c02043_si_001.pdf [file se0c02043_si_001.pdf]

Supporting Information

Conformation and quantum-interference-enhanced thermoelectric properties of diphenyl-diketopyrrolopyrrole (DPP) derivatives.

Renad Almughathawi[1], Songjun Hou[1], Qingqing Wu[1]*, Zitong Liu[2] , Wenjing Hong[3] and Colin Lambert[1]*

[1]Physics Department, Lancaster University, LA1 4YB Lancaster, United Kingdom

[2]Beijing National Laboratory for Molecular Sciences, CAS Key Laboratory of Organic Solids, Institute of Chemistry, Chinese Academy of Sciences, Beijing 100190, China

[3]State Key Laboratory of Physical Chemistry of Solid Surfaces, iChEM, NEL, College of Chemistry and Chemical Engineering, Xiamen University, Xiamen 361005, China

r.almughathawi@Lancaster.ac.uk; q.wu6@lancaster.ac.uk; c.lambert@lancaster.ac.uk

Contents

I. DPP2-connectivity 2

II. DPP3-connectivity 4

III. Tight-binding model-based transmission functions with several values of the sulfur site energy. 6

IV. Orientation of the two methyl groups (-CH_3). 7

V. Molecular orbitals of DPP-isomers with different connectivities 8

VI. Molecular orbitals of thiophene dimers with different connectivities. 9

VII. DPP-isomer thermoelectric properties. 10

VIII. Thermoelectric properties including phonons. 11

IX. Thermoelectric properties for the difuranyl-DPP+TCNQ complex. 12

X. Dihedral angle effect between thiophene ring and DPP core. 12

I. DPP2-connectivity

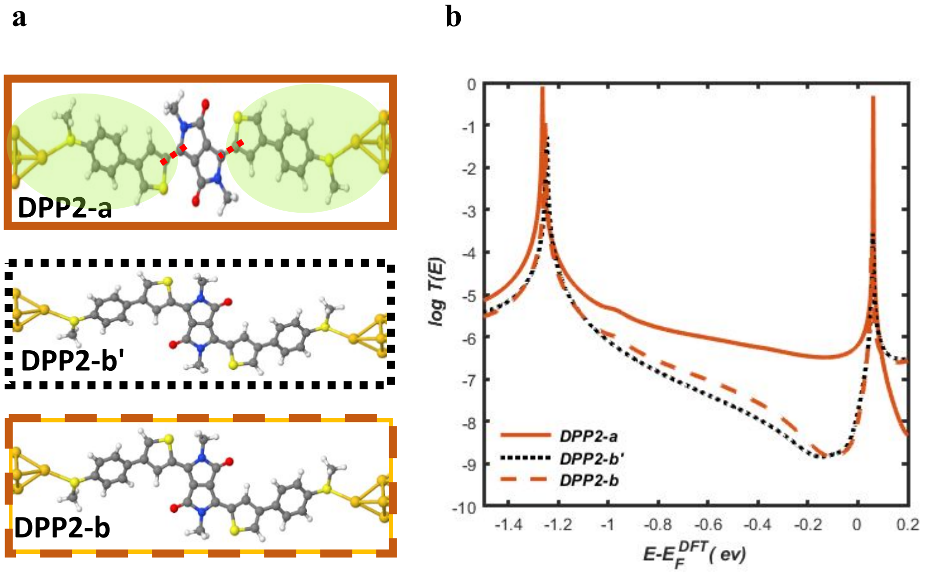

Figure S1. **DFT-based transmission functions for DPP2-a and DPP2-b connected to the gold via SMe-anchor groups.** (a) DPP2-based molecular structures, (b) Transmission coefficients as a function of Fermi-energy for DPP2-a (red solid curve), DPP2-b' (black dotted curve), which is the rotated geometry obtained from DPP2-a and DPP2-b (red dashed curve).

The result in figure S1 shows that after relaxation, the geometries of DPP2-a and DPP2-b have different angles contact between the linker and the electrode. Therefore to test if this could cause the difference in their transmission coefficients (shown as red solid and dashed curves respectively) the transmission coefficient of geometry DPP2-b' (black dotted curve) is shown. The latter geometry is obtained by rotating linker and electrode (indicated by the green shaded regions) relative to the DPP-core through $180°$ degrees around the axis (indicated by the red dashed lines), without further relaxation. Since DPP2-b' and DPP2-b possess similar transmission coefficients, this shows that the $180°$ rotation is the main difference between DPP2-a and DPP2-b. Other geometry changes due to further relaxation, which lead to slight differences between DPP2-b and DPP2-b' have a negligible effect. The corresponding frontier orbitals of the individual gas-phase molecules are shown in table S1.

Table S1: **HOMO, LUMO and LUMO+1 of fully optimised gas-phase molecules DPP2-b, DPP2-b' and DPP2-a, along with their MO energies.**

	**2a(red solid line)**	**2b'(black dotted line)**	**2b(red dashed line)**
**Structures**	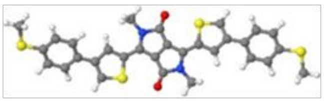	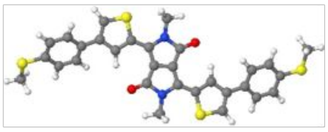	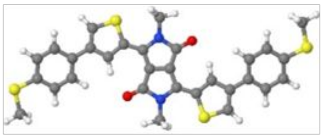
LUMO+1	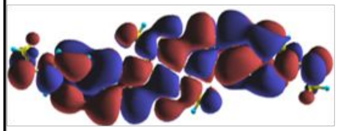	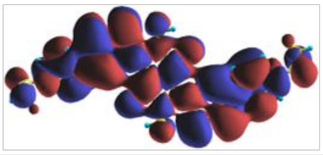	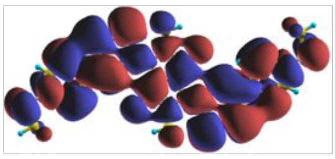
E(eV)	-1.70	-1.70	-1.72
LUMO	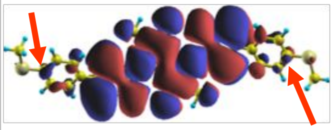	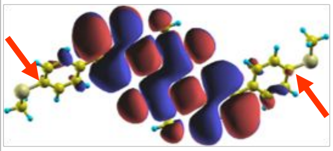	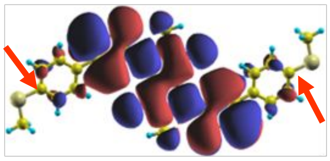
E(eV)	-2.86	-2.90	-2.89
E_F(eV)	-3.55	-3.54	-3.57
HOMO	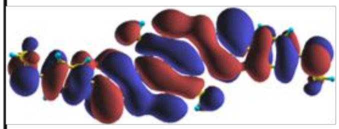	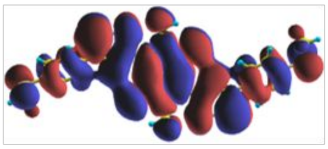	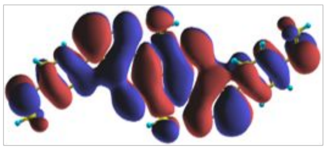
E(eV)	-4. 19	-4.20	-4.20

II. DPP3-connectivity

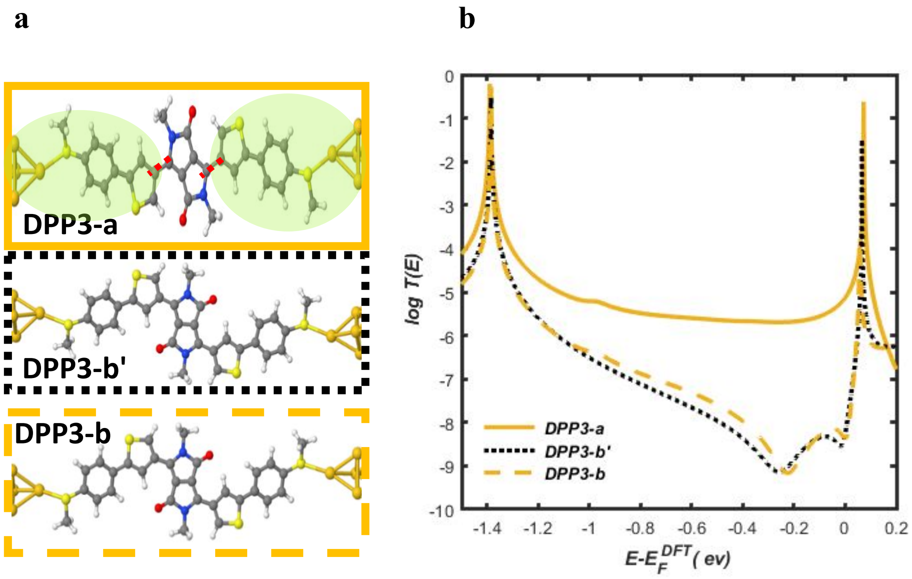

Figure S2**. DFT-based transmission functions for DPP3-a, b connected to the gold via SMe-anchor**. (a) DPP3 based molecular structures, (b) Transmission coefficients as a function of Fermi-energy for DPP3-a (solid curve), DPP3-bꞌ (black dotted curve) and DPP3-b (dashed curve).

Figure S2 shows that after relaxation, the geometries of DPP3- a and DPP3-b have different contact angles between the linker and the electrode. Therefore to test if this could cause the difference in their transmission coefficients (shown as solid and dashed curves respectively) the transmission coefficient of geometry DPP3-bꞌ (black dotted curve) is shown. This geometry is obtained from DPP3- a, by rotating the linkers and their electrodes (indicated by the shaded regions) relative to the DPP-core by $180°$ degrees around the axes show as red dashed lines, without further relaxation. Since DPP3-bꞌ and DPP3-b possess similar transmission coefficients, this shows that a $180°$ rotation is the main reason for the differences in the transmission coefficients between DDP3-a and DPP3-b. The corresponding frontier orbitals of the individual gas-phase molecules are shown in table S2.

The red arrows in tables S1 and S2 show that the LUMO of DPP2-a and DPP3-a have a significant weight on the carbon atoms of the terminal phenyl ring, which bond to the SMe anchor groups, whereas the corresponding weights of the LUMOs of DPP2-b and DPP3-b on these atoms are negligible. Consequently the LUMOs of DPP2-a and DPP3-a contribute to transport, whereas the LUMOs of DPP2-b and DPP3-b do not.

Table S2: **HOMO, LUMO and LUMO+1 of fully optimised gas-phase molecules DPP3-a, DPP3-b' and DPP3-b, along with their MO energies.**

	**3a (yellow solid line)**	**3b'(black dotted line)**	**3b(yellow dashed line)**
**Structures**	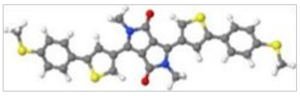	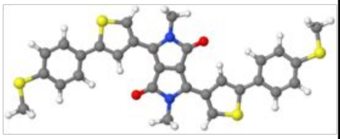	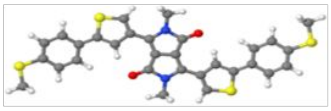
LUMO+1	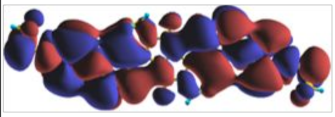	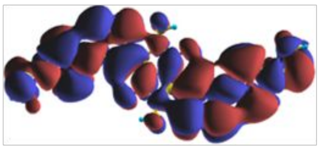	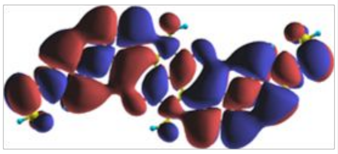
E(eV)	-1.82	-1.73	-1.78
LUMO	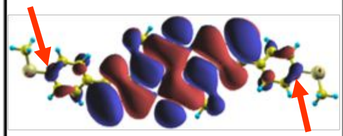	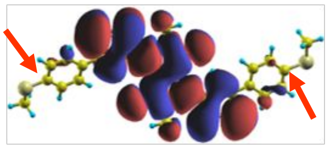	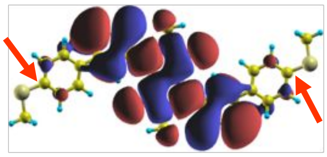
E(eV)	-2.73	-2.82	-2.84
E_F(eV)	-3.472	-3.476	-3.476
HOMO	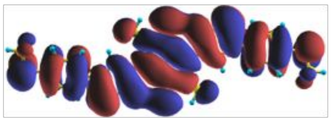	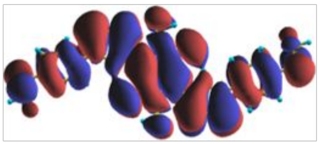	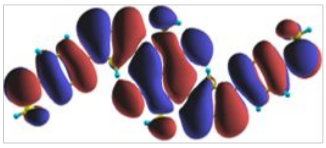
E(eV)	-4.17	-4.26	-4.27

III. Tight-binding model-based transmission functions with several values of the sulfur site energy.

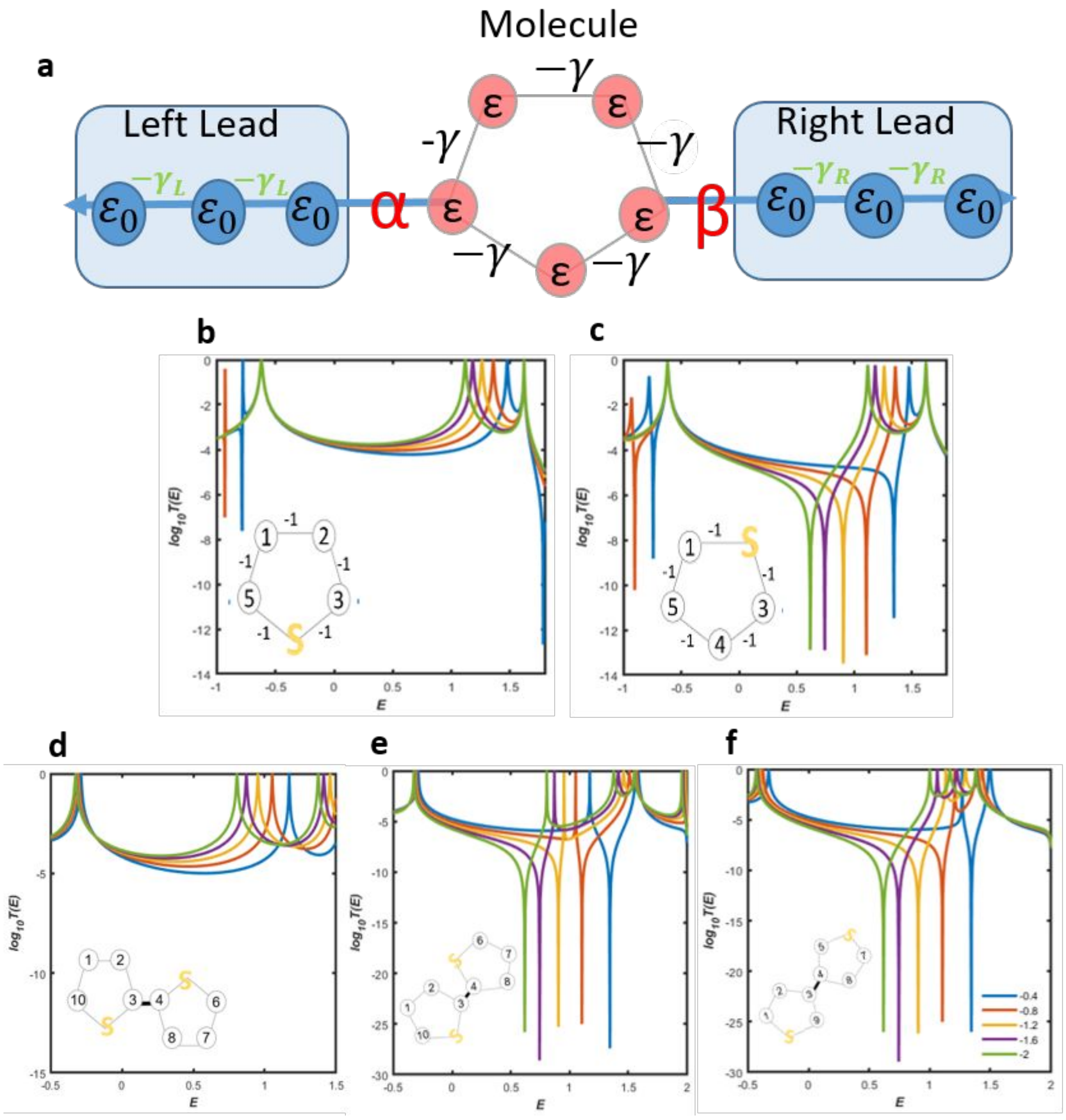

Figure S3. **Tight-binding model-based transmission functions for different connectivities**. (a) A tight binding model consisting of a five-membered ring attached to two semi-infinite one-dimensional chains through weak couplings $\alpha = \beta = 0.1$. All on-site energies of the molecule (red dots) and the leads (blue dots) are set to zero, except those for sulphurs (see Methods). The hopping integrals between two nearest neighbour atoms are set to $-\gamma = -\gamma_L = -\gamma_R = -1$. (b, c) Transmission functions for one thiophene ring with sites 3 and 5 are connected to leads. For each connectivity, several on-site energy values (-0.4, -0.8, -1.2, -1.6, -2) are chosen for the sulfurs in the thiophene rings and the corresponding transmission curves are plotted. (d-f) Transmission functions for two thiophene rings when sites (10, 6) and (1, 7) are connected to leads respectively.

IV. Orientation of the two methyl groups (-CH_3).

After we demonstrate the short molecule with thiophene dimers **S1', S2', S3'** in figure.2b, then we rotate the two methyl groups to be out of the plane. This shows that the electrical conductance affected by the angle of the methyl group bound to the electrode.

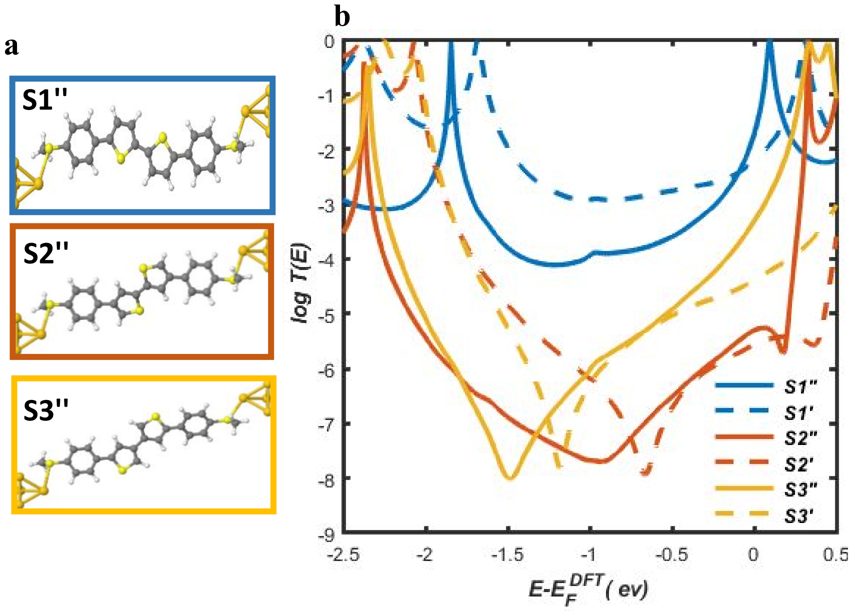

Figure S4. **DFT-based transmission functions for a thiophene-dimer with various SMe rotation angle of the three different connectivities.** (a) Junctions formation of the thiophene-dimer core connected to the gold via SMe out of the plane. (b) the dash curves show the transmission coefficients for the thiophene-dimer, where the SMe in the plane with the core for the three different connectivities **S1', S2', S3'** in Figure 2c. The solid curves show the transmission coefficients for the thiophene-dimer **S1'', S2'', S3''** when the SMe connected to the gold at 90°.

Figure S4 shows that the transmission coefficients depend on the contact angle geometry, for the CQI case in **S1'', S1'** the electrical conductance is higher when the two methyl groups remain in the plane. By contrast, rotating the two methyl groups out of the plane decreased the electrical conductance, while **S2'', S3''** show the opposite behaviour.

V. Molecular orbitals of DPP-isomers with different connectivities

Table S3. **Molecular orbitals of DPP-isomers with different connectivities, along with their MO energies.**

	DPP1-a	DPP1-b	DPP2-a	DPP2-b	DPP3-a	DPP3-b
	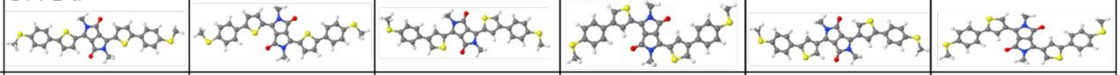					
E_F (eV)	-3.418	-3.439	-3.555	-3.577	-3.472	-3.476
LUMO+2	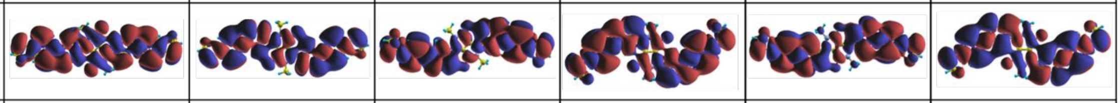					
E(eV)	-1.391	-1.418	-1.445	-1.453	-1.766	-1.688
LUMO+1	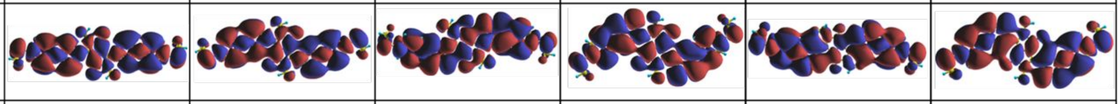					
E(eV)	-1.921	-1.949	-1.700	-1.727	-1.826	-1.782
LUMO	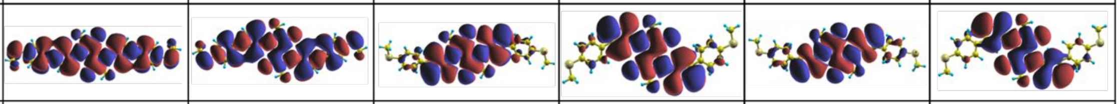					
E(eV)	-2.850	-2.859	-2.866	-2.890	-2.731	-2.846
HOMO	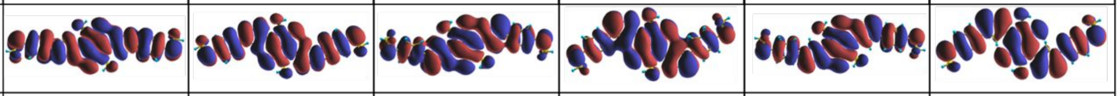					
E(eV)	-3.981	-4.020	-4.197	-4.206	-4.173	-4.277
HOMO-1	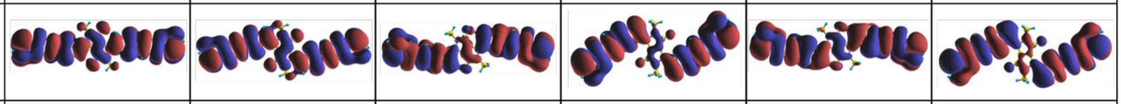					
E(eV)	-4.774	-4.754	-4.695	-4.591	-4.688	-4.530
HOMO-2	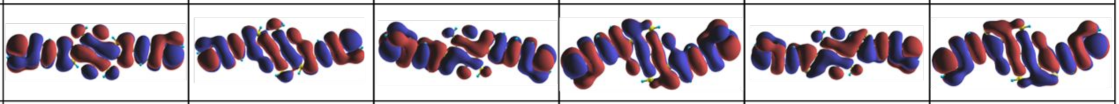					
E(eV)	-4.867	-4.858	-4.729	-4.622	-4.739	-4.579

VI. Molecular orbitals of thiophene dimers with different connectivities.

Table S4. **Molecular orbitals of thiophene dimers with different connectivities, along with their MO energies.**

	DPP1-b 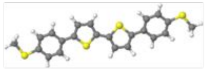	DPP2-a 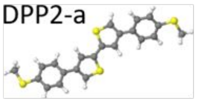	DPP2-b 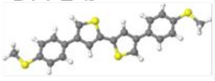	DPP3-a 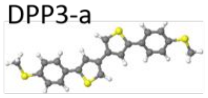	DPP3-b 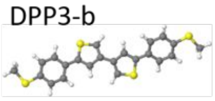
E_F(eV)	-3.16	-3.041	-3.044	-2.88	-2.77
LUMO+2	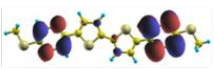	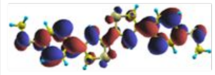	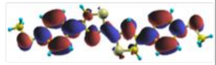	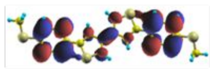	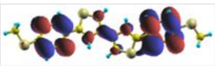
E(eV)	-1.04	-1.13	-1.08	-1.08	-1.06
LUMO+1	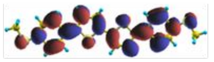	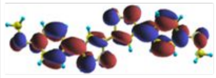	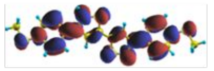	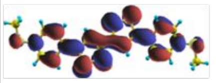	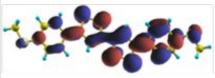
E(eV)	-1.36	-1.34	-1.33	-1.56	-1.63
LUMO	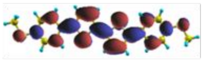	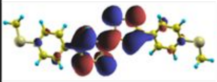	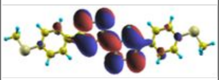	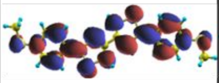	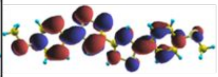
E(eV)	-2.16	-1.80	-1.82	-1.65	-1.69
HOMO	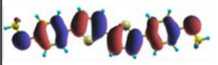	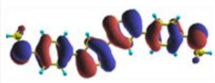	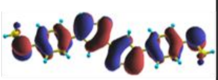	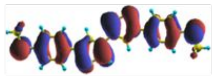	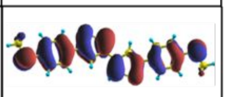
E(eV)	-4.06	-4.358	-4.352	-4.36	-4.33
HOMO-1	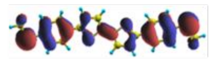	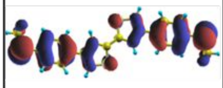	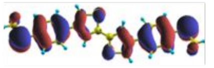	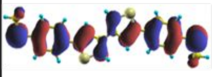	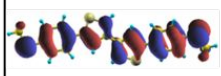
E(eV)	-4.66	-4.545	-4.547	-4.49	-4.46
HOMO-2	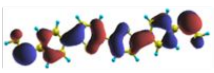	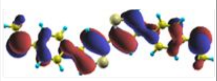	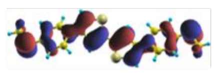	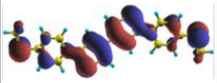	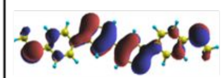
E(eV)	-5.18	-4.892	-4.875	-5.02	-5.07

VII. DPP-isomer thermoelectric properties.

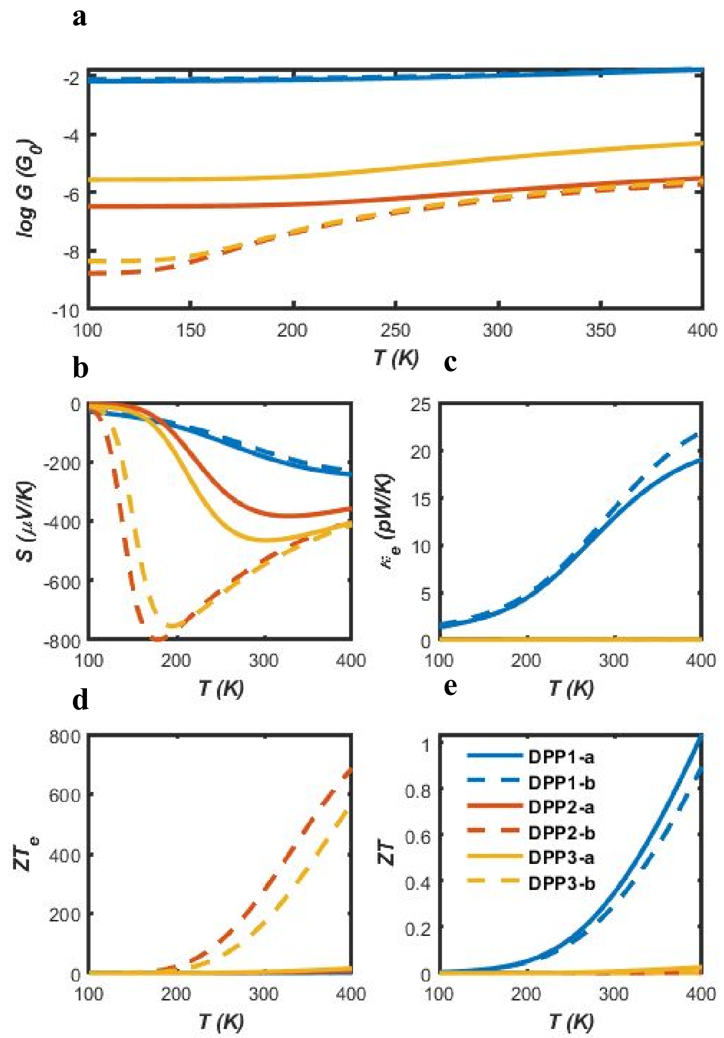

Figure S5. **Thermoelectric properties of the molecules as a function of temperature for DPP- derivatives**: (a-e) the electrical conductance G, Seebeck coefficients $S(T)$, thermal conductance κ_e, electronic figure of merit $\mathrm{ZT}_e(T)$ and full ZT as a function of temperature at Fermi-energy $E_F = -0.1$ respectivly.

VIII. Thermoelectric properties including phonons.

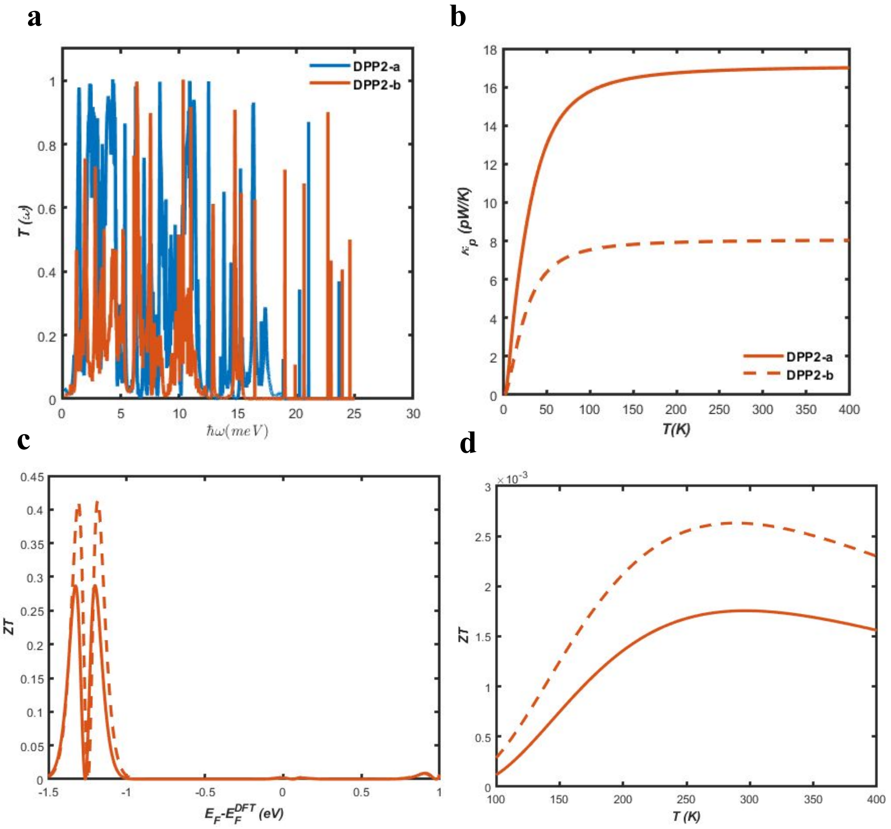

Figure S6. **Thermoelectric properties of the molecules DPP2-a and DPP2-b** (a) phonon transmission function, (b) phononic contribution to the thermal conductance, (c) full ZT as a function of Fermi energy at room temperature $300\ K$, (d) full ZT as a function of temperature.

IX. Thermoelectric properties for the difuranyl-DPP+TCNQ complex.

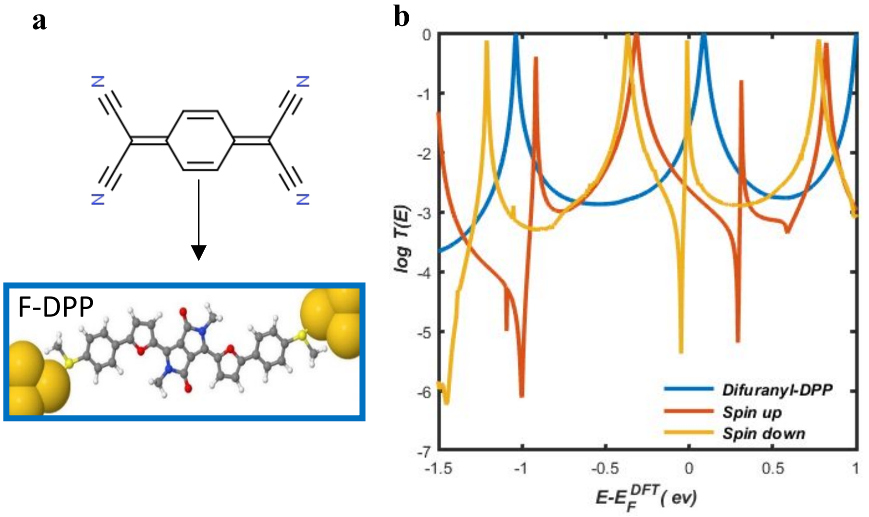

Figure S7. **DFT-based transmission functions of Difuranyl-DPP with TCNQ**. (a,b) An example of an optimized configuration of the system containing a single molecule Difuranyl-DPP (F-DPP) with TCNQ. (b) Transmission coefficients against Fermi energy E_F. Blue curve represents the transmission functions of F-DPP while red and yellow curves depict the spin up and spin down transmission functions of the donor-acceptor charge-transfer complex respectively.

X. Dihedral angle effect between thiophene ring and DPP core.

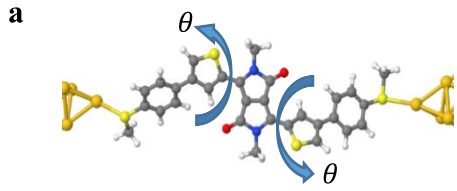

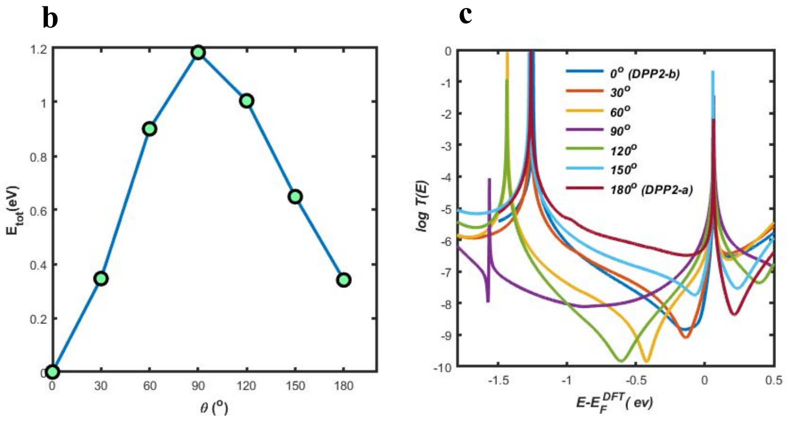

Figure S8: **DFT-based transmission functions for DPP2-a, b connected to the gold via SMe-anchor at different dihedral angles**. (a) For DPP-2, this shows the dihedral angle θ, which is varied from 0° to 180°. (b) Total energy versus θ. (c) Transmission coefficients as a function of energy for DPP2-b molecule at various dihedral angles θ.
